# Multi-task feature integration and interactive active learning for scene image resizing

**DOI:** 10.1038/s41598-025-98917-w

**Published:** 2025-05-19

**Authors:** Ludan Shi, Xianhua Yan, Sen Wang

**Affiliations:** 1 Aeronautical Engineering College, Jinhua University of Vocational Technology, Jinhua, 321007 Zhejiang People’s Republic of China; 2 Attribute Integration Department, Zhejiang smart Intelligence Technology Co., Ltd., Binhai 4th Road 859, Hangzhou Bay, Ningbo, Zhejiang People’s Republic of China; 3 College of Computer Sciences, Beijing Technology And Business University, Beijing, People’s Republic of China

**Keywords:** Resizing, Feature fusion, Multi-task, Active learning, Local preservation, Mathematics and computing, Computer science

## Abstract

In the realm of artificial intelligence (AI), recomposing the semantic segments of intricate scenes is pivotal. This study attempts to seamlessly combine multi-channel perceptual visual features for the adaptive retargeting of images characterized by complex spatial configurations. The key of our approach is the formulation of an in-depth hierarchical model dedicated to the precise capture of human gaze dynamics. Utilizing the BING objectness metric, we swiftly and accurately acquire patches within scenes that hold semantic and visual significance by identifying objects and their components across varying scales. Subsequent to this, we introduce a multi-task feature selector for the dynamic integration of multi-channel features across disparate scene patches. To capture human perception in recognizing critical scenic patches, we introduce a strategy known as locality-preserved and interactive active learning (LIAL). This technique incrementally crafts gaze shift paths (GSP) for each scene. The primary advantages of LIAL are twofold: firstly, it maintains the local coherence of varied scenes efficiently, and secondly, it allows for the active selection process to be shaped by human interaction. By employing LIAL, we methodically represent a GSP for every scene and calculate its corresponding deep features by a multi-layer aggregating algorithm. The deeply-learned GSP representations are subsequently encoded to a Gaussian mixture model (GMM), serving as the basis for scenic image retargeting. Our empirical analyses affirm the effectiveness of our proposed methodology. Statistics of our designed user study showed that our retargeting outperforms the five counterparts. Besides, compared to other 17 popular visual recognizers, our method’s precision exceeds the second best performer by 3%, and the testing time consumption is only 49.8% of the second best performer.

## Introduction

With the continuous evolution of mobile technology, photo retargeting has emerged as a dynamic field. Consider the scenario of transforming a high-resolution $4000\times 2800$ photograph, captured with a DSLR camera, into a $640\times 900$ wallpaper suitable for an iPhone display. This task is fraught with challenges, primarily due to the marked disparity in aspect ratios between the source and target images, which often results in unsatisfactory retargeting characterized by uneven scaling. Simple cropping is inadequate when key compositional elements are spread throughout the image. To achieve optimal retargeting results, modern methods largely rely on content-aware photo retargeting ^9–12,17–19^, which aims to preserve areas of visual significance while minimizing the impact of less important regions. However, these advanced content-aware techniques face two significant challenges:

- Our investigation reveals that high-resolution scenes abound with captivating objects and their components, highlighted in the first section of Fig. 2. Assigning semantic labels to each scene demands a biologically-motivated approach that can emulate human perception in recognizing visually striking regions. The development of a deep learning framework capable of accurately identifying these pivotal areas and enhancing their visual appeal presents several challenges, including: i) determining the sequence of human attention towards appealing segments within an image, as demonstrated by Gaze Shift Paths (GSPs); ii) filtering out extraneous labels in extensive training datasets; and iii) translating semantic labels from an overall image context to specific patches within a scene.
- Semantically or visually significant parts of a scene are typically represented by various low-level descriptors, each providing a unique perspective on the scene through different channels. The amalgamation of these low-level features requires a precise evaluation of the importance of each feature channel. However, crafting an effective strategy to harmonize these channels introduces its own set of challenges, including i) merging local features from spatially adjacent regions within the scene; ii) preserving a comprehensive feature composition throughout different regions of the scene; and iii) adaptively adjusting feature channel weights for diverse sets of scenic images.

**Figure 1:**
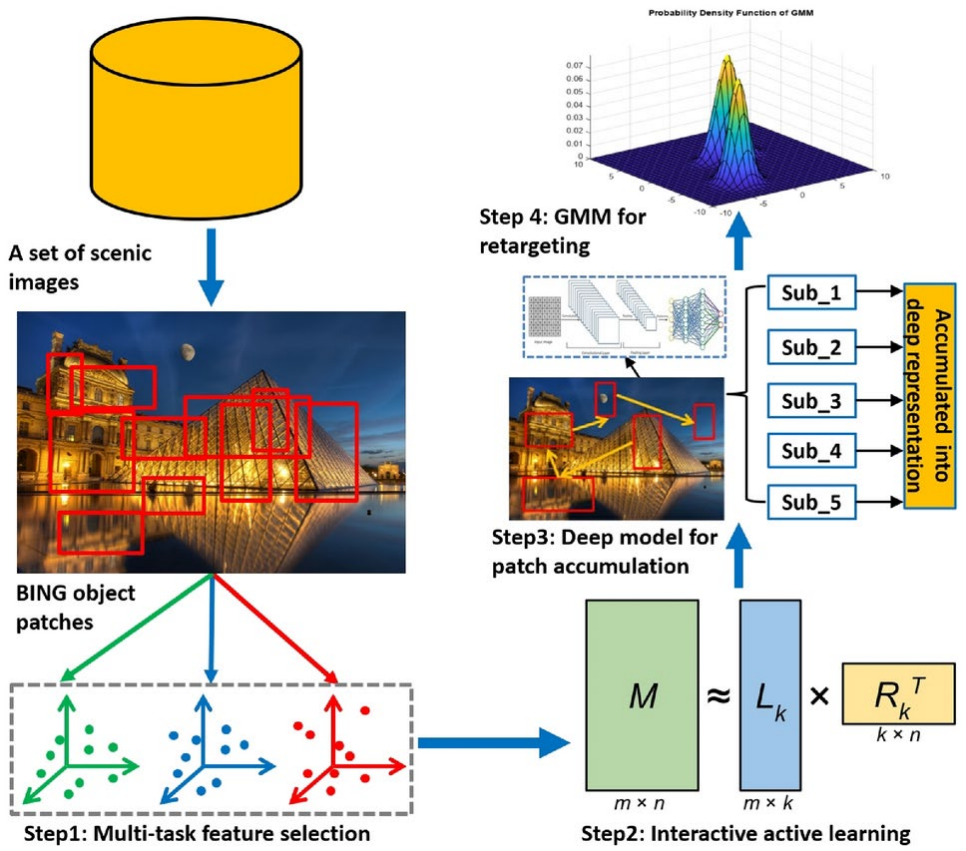
Pipeline of our scene retargeting model.

To tackle the identified challenges, we’ve crafted a pioneering framework for retargeting scenery that closely stimulates human gaze behavior, ensuring an efficient selection of various low-level features for depicting each scene patch. The pipeline is shown in Fig. 1 Confronted with a wide array of scenic images, potentially marked by image-level labels, our initial approach employs the binarized norm gradients (BING) technique to segregate numerous object-focused patches. Following this, we implement a strategy for multi-task selecting low-level features that captures both the underlying structures of these patches. Moreover, to mimic the dynamics of human gaze in scene observation, we introduce a locality-preserved and interacitve active learning (LIAL) model. This model not only identifies the human gaze shift path (GSP) but also preserves the local coherence of the scene. By leveraging the deep GSP features obtained using an deep aggregation model, we incorporate visually appealing scenic images into a Gaussian mixture model (GMM), thereby facilitating the retargeting process. The effectiveness of our approach is validated through extensive empirical assessments across a variety of scenic images and a comprehensive user study.

This work’s novelties are two-fold: Firstly, the **LIAL framework** (Locality-Preserved and Interactive Active Learning) actively captures gaze-informed visual features, enabling human interactions to be intrinsically incorporated into the scene resizing process. Unlike other methods, which cannot explicitly calculate or use human gaze allocations, our approach aligns with natural human perception by modeling how individuals prioritize and engage with specific regions of a scene. This enhances scene understanding and provides a more intuitive, human-centric way of resizing scenes, offering greater interpretability and relevance. Secondly, we introduce a **multi-task feature selection method** that dynamically measures the relevance of different feature channels for each scenic patch. This allows the model to optimize feature discrimination in real time, ensuring that selected features are globally discriminative across all semantic labels. In contrast, traditional feature selectors often fail to simultaneously optimize for multiple labels, limiting their effectiveness in complex scenes with overlapping categories. Our method, therefore, ensures that features are not only informative for individual patches but also discriminative across the entire scene, offering more robust and nuanced scene categorization. These two innovations-incorporating human gaze dynamics and enabling dynamic, multi-label optimization-set our method apart from existing techniques, improving accuracy, adaptability, and interpretability for real-world applications.

The rest parts of this article are organized as: Section “Previous techniques” reviews relevant studies. Section “Scenery interpretation technique” describes our framework for retargeting scenic photos, including three key components: (1) integrating features across multiple tasks for scenic patch depiction, (2) applying the LIAL method for generating Gaze Shift Paths (GSPs), and (3) using a GMM for the retargeting of scenic images. Section “Empirical assessment” presents empirical evidence demonstrating the enhanced performance of our approach. Section “Conclusions” concludes the article.

## Previous techniques

### Deep scene categorization models

In the domain of computer vision, remarkable strides have been made with the advent of deep scene categorization models, particularly those employing hierarchically organized Convolutional Neural Networks (CNNs) and sophisticated deep learning frameworks. These advancements have shown exceptional ability in scene recognition across extensive image datasets, notably ImageNet ^2^. A significant breakthrough was achieved with the introduction of a deep neural network model trained on a subset of ImageNet images ^2^, which exhibited unparalleled accuracy in scene categorization, as detailed in ^3^. This evolution has significantly broadened the application of ImageNet-CNNs, enriching various vision applications like video parsing and anomaly detection. The evolution of ImageNet-based CNNs has primarily focused on accumulating extensive datasets for effective training and the development of region-level CNN (RCNN) ^5^ to emphasize the importance of quality patch sampling. Despite these advancements, the efficiency of training deep visual models directly with entire scenic images or arbitrary patches remains a challenge. To address this, innovative approaches like the one presented in ^7^ have been employed, utilizing pre-trained hierarchical CNNs to discern significant local scenic patches, thereby enhancing the depth of scene categorization learning. Furthermore, a multi-task and multi-resolution scene categorization algorithm that maintains intrinsic feature distributions through a manifold regularizer was developed in ^26^. Additionally, ^27^ introduced a semantic annotation scheme employing low-rank deep features for accurate category posterior probability estimation, coupled with a Markov model for contextual feature learning. Subsequent research in ^38^ explored the potential of deep layer interrelations and an unlabeled learning model to adjust deep features considering the geometrical attributes of scenes. In a parallel development, ^43^ proposed a novel approach integrating discriminative feature learning with weak label learning in scene analysis, employing a stacked discriminative sparsity autoencoder for deriving high-level visual representations. The deep learning framework presented in^53^ incorporates channel and spatial attention modules in sequence, enhancing feature extraction and temporal dependency modeling with a bi-directional LSTM network. Trained on a tennis dataset, the network employs both cross-entropy and marginal loss functions for action classification, achieving strong precision and recall results across six tennis players’ actions.

While these deep learning models, particularly those based on CNNs, have significantly advanced scene categorization, they primarily focus on dataset size and local patch recognition. In contrast, our method enhances scene categorization by incorporating human gaze dynamics through Gaze Shift Paths (GSPs), offering a more human-centered approach to identifying semantically and visually significant areas within a scene. This innovation allows for better precision and faster training times, overcoming the challenges faced by traditional region-level CNNs and making the learning process more intuitive and efficient. By emulating human visual attention, our method adds an additional layer of interpretability, ensuring that the model not only performs well but also provides more meaningful explanations for its predictions. This approach is particularly beneficial in applications where understanding how a model makes decisions is as important as the final result itself. Moreover, the dynamic integration of multi-channel features through our multi-task feature selector further improves the accuracy of scene recognition, offering a substantial improvement over traditional deep learning methods that may struggle with visual complexity.

### Aerial image categorization

In the aerial image categorization field, cross-modality learning strategies have been introduced, employing pixel and spatial features for classification purposes, as evidenced by the work of Danfeng et al. ^44^. Similarly, the authors ^45^ developed a semantic transforming model specifically designed for aerial picture categorization, capturing long-term relationships across different regions. Chalavadi et al. ^8^ devised mSODANet, a parallel deep learning framework focused on extracting contextual features from ground objects at various scales. These models collectively utilize region-level discriminative cues for scene classification, either in a supervised or unsupervised manner. Despite their effectiveness as demonstrated through empirical studies, these models do not directly incorporate human perceptual insights into the classification framework. Our method distinguishes itself by embedding human gaze sequences into the classification process, providing unique semantic interpretability and leveraging Gaze Shift Paths (GSPs), thus offering a distinct competitive edge over existing techniques.

While existing methods in aerial image categorization focus on pixel and spatial features or cross-modality learning, they lack human perceptual insights in their classification processes. Our method distinguishes itself by embedding human gaze sequences into the classification framework, incorporating semantic interpretability into scene categorization. This provides not only higher accuracy but also a more intuitive understanding of how scenes are categorized, setting our approach apart from previous models in both interpretability and performance. By focusing on human-like attention patterns, our method is more aligned with how humans naturally interpret and prioritize visual information, making it more adaptable to real-world applications where human input is often essential. Additionally, the incorporation of Gaze Shift Paths (GSPs) allows for a more precise extraction of meaningful features from aerial images, enhancing the model’s ability to handle complex, multi-scale scenes with greater efficiency. This capability makes our method particularly well-suited for real-time classification tasks in dynamic environments, where traditional models may struggle due to their lack of perceptual grounding.

Zhang et al. ^69^ proposed an adaptive differentiation Siamese fusion network to enhance remote sensing change detection by effectively handling spatial and temporal changes. Zhang et al. ^70^ introduced an asymmetric light-aware progressive decoding network that improved RGB-thermal salient object detection by addressing varying light conditions. Zhang et al. ^71^ presented CFANet, an efficient method for UAV image detection based on cross-layer feature aggregation, optimizing the detection of features from aerial images. Zhang et al. ^72^ proposed VSS-Net, a visual semantic self-mining network designed for video summarization by extracting meaningful semantic information from video content. Zhang et al. ^73^ introduced a full-scale feature aggregation and grouping feature reconstruction method for UAV image target detection, which enhanced detection performance across different scales. Finally, Zhang et al. ^74^ developed a multi-scale spatiotemporal feature fusion network for video saliency prediction, improving the prediction of salient regions in video frames.

### Image resizing and scene resizing

Image retargeting, also known as image resizing or content-aware image resizing, refers to the process of adjusting the size or aspect ratio of an image while attempting to preserve its most important content and visual features. Traditional methods for image resizing often rely on heuristic approaches, which typically focus on removing or adding pixels in a way that attempts to retain the perceived importance of different areas of the image. One of the earliest and most influential methods in this field is seam carving, proposed by Avidan and Shamir ^28^. Seam carving uses dynamic programming to identify and remove or insert seams (paths of low energy) in the image, allowing for non-uniform resizing while preserving visually significant areas. While this approach works well for certain types of images, it struggles with content that is highly structured or that has significant objects spanning multiple rows or columns. Other popular approaches to image retargeting include mesh-based methods, which divide the image into a mesh of regions and resize each region based on its importance. One such method is the image retargeting approach based on graph cuts, where a global energy minimization framework is employed to minimize distortion during resizing ^29^. These techniques generally offer improvements in preserving object shapes and structure during resizing. Mesh-based methods are often guided by feature maps that indicate the importance of different regions in the image, such as edges, texture, or saliency ^30^. Despite their successes, they often struggle with maintaining consistent visual coherence across different image types, particularly when there is significant variation in content or the presence of multiple objects in a scene.

In more recent works, learning-based methods have emerged as a promising direction for image retargeting. One example is the method proposed by Zhang et al. ^31^, which employs deep learning models to predict image saliency and adjust the resizing process accordingly. By training a convolutional neural network (CNN) on large datasets, these methods learn to identify key regions in an image that should be preserved during retargeting. This approach significantly outperforms traditional heuristic methods in terms of image quality and consistency, especially in images containing complex structures or diverse objects. More recently, generative approaches have been used for image retargeting. For instance, the use of generative adversarial networks (GANs) for image resizing has been proposed by Bao et al. ^32^, where the network learns to generate retargeted images that preserve both spatial content and perceptual quality. These methods are capable of handling highly dynamic content and complex structures, offering an advanced alternative to traditional resizing techniques. Recent advancements beyond 2020 have further enhanced deep learning-based retargeting techniques. For example, a deep image retargeting method using a dual-branch convolutional neural network (CNN) was proposed by Liu et al. ^34^, which effectively preserves important image structures during the retargeting process while also reducing computational cost. Their dual-branch architecture learns spatial and semantic features separately and merges them for effective retargeting, achieving better preservation of both local content and global layout.

Another significant development came from the work of Zhang et al. ^35^, who introduced a novel content-aware image retargeting model based on attention mechanisms. Their method uses an attention module to selectively focus on regions of high visual importance, ensuring that these areas are preserved during resizing. This method shows significant improvements in preserving fine details in complex scenes, especially in images with objects that occupy a large portion of the image, such as people or landscapes. The introduction of attention mechanisms allowed the model to dynamically adjust the focus of the retargeting process, leading to better visual results. In 2022, Li et al. ^36^ proposed an image retargeting framework that combines a convolutional neural network with reinforcement learning (RL). This hybrid model learns to adjust the retargeting process in a more flexible manner by using a reinforcement learning agent that fine-tunes the resizing parameters based on reward signals that reflect perceptual quality. This approach offers a more adaptive solution to retargeting and achieves superior performance over previous methods in terms of both visual quality and efficiency. In addition, a multi-scale approach was introduced by Yang et al. ^37^ in 2023, which utilizes multi-scale learning to better capture the hierarchical nature of content in images. Their approach integrates multi-scale deep learning to refine resizing at multiple levels of detail, improving retargeting accuracy, especially in images with high complexity or intricate details. The multi-scale method enables the network to focus on both coarse and fine details of the image, achieving a high-quality result in resizing tasks. However, these methods often struggle with videos, where temporal consistency is just as important as spatial consistency. Video retargeting introduces additional challenges, as the resized frames must align temporally, maintaining the smooth flow of motion across successive frames. A promising solution to this problem involves incorporating spatiotemporal learning models that learn both spatial and temporal features from videos, such as the work by Tran et al. ^33^ with 3D CNNs, which effectively capture the temporal dynamics of videos while preserving key content across frames.

The key takeaway from these advancements is that while traditional methods rely on fixed, manually defined heuristics or simple optimizations, modern approaches leverage deep learning to better understand the content and context of an image, resulting in more natural and adaptive resizing. Learning-based methods, particularly those involving CNNs or GANs, allow for automatic adaptation to different types of content, enabling the preservation of important features while avoiding common issues such as distortion or content loss. Traditional image resizing and retargeting algorithms often rely on heuristic models and deep aesthetic judgments, which can lead to suboptimal results. Our approach improves upon these by learning Gaze Shift Paths (GSPs) directly from a diverse set of photographs. This human-centered learning process ensures that retargeting is more visually compelling and perceptually relevant, delivering superior results compared to conventional methods. Additionally, our method enhances fine-grained recognition in video content and addresses class imbalance issues more effectively, which traditional approaches do not fully tackle.

## Scenery interpretation technique

Herein, we delineate the architecture of the proposed technique for scenic image retargeting, which is segmented into four integral parts. The first part details the process for extracting scenic patches that are both semantically and visually significant. Following this, we explore the dynamic integration of features at the patch level in the second component. The third part delves into our tailored LIAL algorithm, specifically aimed at the active selection of scenic patches guided by gaze patterns to formulate Gaze Shift Paths (GSPs). The culmination of our framework is discussed in the fourth component, where we elaborate on the application of a Gaussian Mixture Model (GMM) to adeptly retarget scenic images. This holistic approach ensures a comprehensive treatment of scenic images, from identifying key patches to the final retargeting process, encapsulating the core elements necessary for effective image interpretation and adaptation.

### Identifying scenic patches

Studies in visual cognition and psychology ^22,23^ have shown a consistent focus of human gaze on semantically or visually significant areas within a scene, underscoring the human tendency to pay attention to specific, distinctive regions. This insight forms the basis of our approach to scene categorization, where we integrate the detection of object-centric patches through the Locality-preserved and Interactive Active Learning (LIAL) method, aiming to identify those crucial scenic patches that align with human visual preferences.

In real-life scenarios, humans naturally focus on prominent objects or their components, such as vehicles or skyscrapers, which stand out due to their importance and placement within the scene. To effectively identify objects or components likely to capture human interest, we utilize the BING ^1^ objectness measure, a method renowned for its efficiency in extracting a collection of high-quality, object-oriented patches from diverse scenes, which we term “scenic patches.” The advantages of employing the BING method are threefold: it is highly efficient in detecting object patches with minimal computational demands; it significantly aids in the extraction of Gaze Shift Paths (GSP) by providing a superior array of object-level patches; and it exhibits an excellent capacity to generalize to unidentified object categories, thereby enhancing the adaptability of our scene categorization framework across various datasets.

### Multi-task feature integration from patches

After extracting object-related patches from scenic images via the BING technique ^1^, we gather a collection of low-level features from each patch. Following this, a multi-channel feature fusion algorithm is implemented to effectively merge these features. This method employs a combined multi-task approach, which is detailed in the following:

Mathematically, our multi-task feature selector’s objective is to identify features toward *u* different tasks (in our implementation, *u* means the number of different labels). The *m*-th task corresponds to the $t_m$ scenic images $\{x_m^1,x_m^2,\cdots , x_m^{t_m}\}$ combined with the pre-specified labels $\{y_m^1,y_m^2,\cdots , y_m^{t_m}\}$ from $c_m$ categories. In our implementation, we simply set $\textbf{X}_m=[x_m^1,x_m^2,\cdots , x_m^{t_m}]$ as the data matrix corresponding to the *m*-th task. Meanwhile, we set $\textbf{Y}_m=[y_m^1,y_m^2,\cdots , y_m^{t_m}]$ as the matrix containing the corresponding labels. More specifically, for a matrix $\textbf{Z}\in \mathbb {R}^{b\times c}$ and *b* and *c* denoting any positive integers, $||\cdot ||_F$ represents the Frobenius norm. Meanwhile, the matrix-level $l_{2,1}$-norm is calculated by:

1 $$\begin{aligned} ||\textbf{Z}||_{2,1}=\sum \nolimits _i \left( \sum \nolimits _j \textbf{Z}_{i,j}^2\right) ^{1/2}. \end{aligned}$$

For the rest of this article, we denote $\text {tr}(\cdot )$ as the trace operation, $\textbf{J}_{t_m}$ denotes the $t_m\times t_m$ identity matrix. Besides, $\textbf{1}_{t_m}$ denotes the column vector wherein the entire elements are all fixed to ones.

Assuming for the *p*-th task, the so-called label indicator matrix $\textbf{G}_p$ is defined as follows: $\textbf{G}_p=\textbf{Y}_p(\textbf{Y}_p^T\textbf{Y}_p)^{-1/2}$. Afterward, to the *p*-th task, the scatter between different classes and that from the entire classes are respectively defined as: $\textbf{T}_b^{(p)}=\bar{\textbf{X}}_p\textbf{G}_p\textbf{G}_p^T\bar{\textbf{X}}_p^T$, and $\textbf{T}_{t}^{(p)}=\bar{\textbf{X}}_p\bar{\textbf{X}}_p^T$, where $\bar{\textbf{X}}_p=\textbf{X}_p\textbf{K}_l$ and $\textbf{K}_l=\textbf{I}_{m_l}-\textbf{1}_{m_l}\textbf{1}_{m_l}^T/{m_l}$ denotes the matrix of the center samples. By leveraging the mechanism of LDA ^16^, we can formulate the following task-dependent feature selector:

2 $$\begin{aligned} \min _{\textbf{U}_p^T\textbf{U}_p=\textbf{I}|_{p=1}^u}\sum _{p=1}^t \frac{\text {tr} (\textbf{U}_p^T\bar{\textbf{X}}_l(\textbf{I}_{m_l}-\textbf{G}_p\textbf{G}_p^T)\bar{\textbf{X}}_l^T\textbf{U}_l)}{\text {tr}(\textbf{U}_l^T\bar{\textbf{X}}_l\bar{\textbf{X}}_l^T\textbf{U}_l)}\nonumber \\ +\alpha _1\sum _i(\sum _j(\textbf{U}_{i,j}^{(p)})^2)^{p/2}+\alpha _2\sum _i(\sum _j \textbf{U}_{i,j}^2)^{1/2}, \end{aligned}$$

Herein, the symbols can be detailed in the following: $\alpha _1,\alpha _2$ denotes the weights of two regularizers, $\textbf{U}_{i,j}^{(j)}$ denotes (*i*, *j*)-th entity from the conversion matrix $\textbf{U}_p$ with respect to the *p*-th task. Meanwhile, $\textbf{U}=[\textbf{U}_1,\textbf{U}_2,\cdots , \textbf{U}_u]$ represents the combined selection matrix corresponding to the entire tasks. To our best knowledge, the above equation (2) is optimized using an iterative algorithm, that is, we iteratively calculate $\textbf{U}_p$ ($p=1,2,\cdots ,u$) until convergence.

Denoting $\textbf{F}_p=\bar{\textbf{X}}(\textbf{I}_{m_l}-\textbf{G}_p\textbf{G}_p^T)\bar{\textbf{X}}_p^T$ and $\textbf{C}_p=\bar{\textbf{X}}_p\bar{\textbf{X}}_p^T$ and fix $\textbf{U}_j$ ($j=1,\cdots , p-1, p+1,\cdots ,u$), the above objective function is reorganized as:

3 $$\begin{aligned} \min _{\textbf{U}_p^T\textbf{U}_p=\textbf{I}} \frac{\text {tr}(\textbf{U}_p^T\textbf{F}_p\textbf{U}_p)}{\text {tr}(\textbf{U}_p^T\textbf{C}_p\textbf{U}_p)}+ \alpha _1\text {tr}(\textbf{U}_p^T\textbf{E}_p\textbf{U}_p)+\alpha _2\text {tr}(\textbf{U}_p^T\textbf{EU}_p), \end{aligned}$$

where $\textbf{E}_p$ and $\textbf{E}$ denote two diagonal matrices, each diagonal entity is calculated as:

4 $$\begin{aligned} e_{ii}^{(p)}=\frac{1}{2||\textbf{u}_p^i||_2}, \end{aligned}$$

5 $$\begin{aligned} e_{ii}=\frac{1}{2||\textbf{u}^i||_2}, \end{aligned}$$

where $\textbf{u}_p^i$ and $\textbf{u}_p$ respectively denote the *p*-th row from matrix $\textbf{U}_p$ and $\textbf{U}$.

In practice, an approximate solution of (3) can be obtained as:

6 $$\begin{aligned} \min _{\textbf{U}_p^T\textbf{U}_u=\textbf{I}} & \hspace{-20pt}\text {tr}(\textbf{U}_p^T(\textbf{F}_p-\beta \textbf{C}_p)\textbf{U}_p)+\alpha _1 \text {tr}(\textbf{U}_p^T\textbf{E}_p\textbf{U}_p)+\nonumber \\ & \hspace{-20pt}\alpha _2 \text {tr}(\textbf{U}_p^T\textbf{EU}_l), \end{aligned}$$

where $\beta$ weights the significance of $\textbf{C}_p$. Generally, it can be approximated as $\beta =\frac{\text {tr}(\bar{\textbf{U}}_p^T\textbf{F}_p\bar{\textbf{U}}_p)}{\text {tr}(\bar{\textbf{U}}_p^T\textbf{C}_p\bar{\textbf{U}}_p)}$ and $\bar{\textbf{U}}_p=\arg \min _{\textbf{U}_p}\frac{\text {tr}(\bar{\textbf{U}}_p^T\textbf{F}_p\bar{\textbf{U}}_p)}{\text {tr}(\bar{\textbf{U}}_p^T\textbf{C}_p\bar{\textbf{U}}_p)}$.

Denoting $\textbf{N}=(\textbf{F}_p-\beta \textbf{C}_p)$, (6) can be computed as:

7 $$\begin{aligned} \min _{\textbf{U}_p^T\textbf{U}_p=\textbf{I}} \text {tr}(\textbf{U}_p^T\textbf{NU}_p)+\alpha _1 (\textbf{U}_p^T(\textbf{E}_p+\pi \textbf{E})\textbf{U}_p), \end{aligned}$$

where $\pi =\alpha _1/\alpha _2$. In this way, the resulting $\textbf{U}$ is iteratively calculated based on (7) corresponding to *l*-th task when convergence criteria is met. If we have obtained $\textbf{U}$, then the entire features are ranked based on $||\textbf{u}^i||_F$ in a descending way. Herein, only the top-ranking features are deemed sufficiently discriminative.

### Detecting gaze-focused scenic patches by LIAL

By integrating multiple low-level features at the patch level, as previously described, encoding human gaze shifting behavior into our scenic resizing framework is crucial. To achieve this, we utilize an innovative active learning approach that simulates the active perception of humans towards various patches within a scene.

In the realm of scenic imagery, a substantial number of patches may lack the necessary descriptiveness for their semantic categories, often representing background elements that fail to draw human attention. To construct an efficient scenery retargeting model, we introduce a LIAL method aimed at identifying semantically enriched image patches within each scene.

Our objective is to apply a machine learning strategy that effectively discerns the sample distribution. Considering that spatially proximate image patches are likely to have semantic relationships, we opt for a linear reconstruction of each patch using its adjacent counterparts. The parameters for this reconstruction are established as follows:

8 $$\begin{aligned} & \mathop {\arg \min }_\textbf{R}\sum \nolimits _{j=1}^{N} \Vert x_i-\sum \nolimits _{i=1}^{N}\textbf{S}_{ij}y_j\Vert \nonumber \\ & subject~to~\sum \nolimits \textbf{S}_{j=1}^N=1, \textbf{S}_{ij}=0~~if~~y_j\notin \mathcal {B}(y_i), \end{aligned}$$

where ${y_1,y_2,\cdots ,y_N}$ are the visual features of *N* image patches, and $\textbf{R}_{ij}$ indicates the significance of each patch in reconstructing its adjacent patch.

To assess the visual descriptiveness of chosen image patches, we develop a reconstructing algorithm based on these parameters. The error in selected image patches is calculated by:

9 $$\begin{aligned} \hspace{-30pt} & \varepsilon (b_1,b_2,\cdots ,b_N)\nonumber \\ \hspace{-30pt} & =\sum \nolimits _{t=1}^{K}\left| \left| b_{s_t}-c_{s_t}\right| \right| ^2+ \mu \sum \nolimits _{t=1}^{N}\left| \left| b_t-\sum \nolimits _{j=1}^{N} \textbf{S}_{tj}b_j\right| \right| ^2, \end{aligned}$$

where $\mu$ is the regularizer’s weight, and *K* represents the count of selected image patches.

Let $\textbf{C}$ is comprised by $c_i$ and $\textbf{B}$ contains $b_i$, we define $\mathbf {\Upsilon }$ as a diagonal matrix with entries set to one for selected patch indices and zero otherwise. The objective function can be updated to:

10 $$\begin{aligned} \varepsilon (\textbf{B})=\text {tr}\left( (\textbf{B}-\textbf{C})^T \mathbf {\Upsilon }(\textbf{B}-\textbf{C})\right) +\mu \text {tr}(\textbf{B}^T\textbf{DB}), \end{aligned}$$

with $\textbf{D}=(\textbf{I}-\textbf{S})^T(\textbf{I}-\textbf{S})$. To optimize this, we set the gradient of $\epsilon (\textbf{B})$ to zero, leading to:

11 $$\begin{aligned} \mathbf {\Upsilon }(\textbf{A}-\textbf{B})+\mu \textbf{DA} = 0. \end{aligned}$$

The reconstructed image patches are thus computed as:

12 $$\begin{aligned} \textbf{B}=(\mu \textbf{D}+\mathbf {\Upsilon })^{-1}\mathbf {\Upsilon } \textbf{C}, \end{aligned}$$

In our approach, we use reconstructed image patches to calculate the reconstruction error as follows:

13 $$\begin{aligned} \hspace{-20pt} & \varepsilon (b_{s_1},\cdots ,b_{s_K})=|\textbf{B}-\textbf{A}|_F^{2}=|\textbf{B}-(\mu \textbf{D}+\mathbf {\Upsilon })^{-1}\mathbf {\Upsilon } \textbf{X}|_F^{2}\nonumber \\ \hspace{-20pt} & =|(\mu \textbf{D}+\mathbf {\Upsilon })\mu \textbf{DX}|_F^{2}, \end{aligned}$$

Given the combinatorial characteristics, optimizing (13) can be computationally challenging. To address this, we introduce a sequential approach. We denote several refined patches within each scenery as $\{c_{s_1},\cdots ,c_{t_{L'}}\}$. $\mathbf {\Upsilon }n$ represents an $N\times N$ diagonal matrix, and $\mathbf {\Gamma }i$ as an $N\times N$ matrix with ones on the diagonal and zeros elsewhere. Thus, the $t_{L'+1}$-th patch is derived using the below objective function:

14 $$\begin{aligned} t_{L'+1}=\mathop {\arg \min }_{i\notin \{t_1,\cdots ,t_{L'}\}}|(\mu \textbf{D}+\mathbf {\Upsilon }_n+\mathbf {\Gamma }_i)^{-1}\mu \textbf{DY}|_{F}^2. \end{aligned}$$

Notably, $\textbf{D}$ in equation (14) is sparse, which allows us to expedite matrix inversion calculations using the Sherman-Morrison formula:

15 $$\begin{aligned} (\mu \textbf{D}+\mathbf {\Upsilon }_n+\mathbf {\Gamma }_i)^{-1} = \textbf{J}-\frac{\textbf{J}_{*i}\textbf{J}_{i*}}{1+\textbf{J}_{ii}}, \end{aligned}$$

where $\textbf{J}{i}$ and $\textbf{J}_{i}$ respectively represent the *i*-th column and row of matrix $\textbf{J}$. Consequently, the objective function in (14) becomes:

16 $$\begin{aligned} & |(\mu \textbf{D}+\mathbf {\Upsilon }_n+\mathbf {\Gamma }_i)^{-1}\mu \textbf{DB}|{F}^2=\mu ^2tr(\textbf{JDBB}^T\textbf{DJ})\nonumber \\ & \quad -\frac{2\mu ^2 \textbf{DBB}^T\textbf{DBB}_{i}}{1+\textbf{J}_{ii}}+\frac{\mu ^2\textbf{J}_{i}\textbf{J}_{*i}\textbf{DBB}^T\textbf{DB}_{*i}}{(1+\textbf{J}_{ii})^2}, \end{aligned}$$

By setting $\textbf{M}=\textbf{DBB}^T\textbf{D}$, we refine the optimization in (14) to:

17 $$\begin{aligned} & t_{L'+1} = \mathop {\arg \min }_{i\notin \{t_1,\cdots ,t_{L'}\}} \frac{1}{1+\textbf{J}_{ii}}(\frac{\textbf{J}_{i*}\textbf{J}_{*i}\textbf{J}_{i*}\textbf{MJ}_{*i}}{1+\textbf{J}_{ii}}\nonumber \\ & -2\textbf{J}_{i*}\textbf{MJJ}_{*i}). \end{aligned}$$

**Figure 2:**
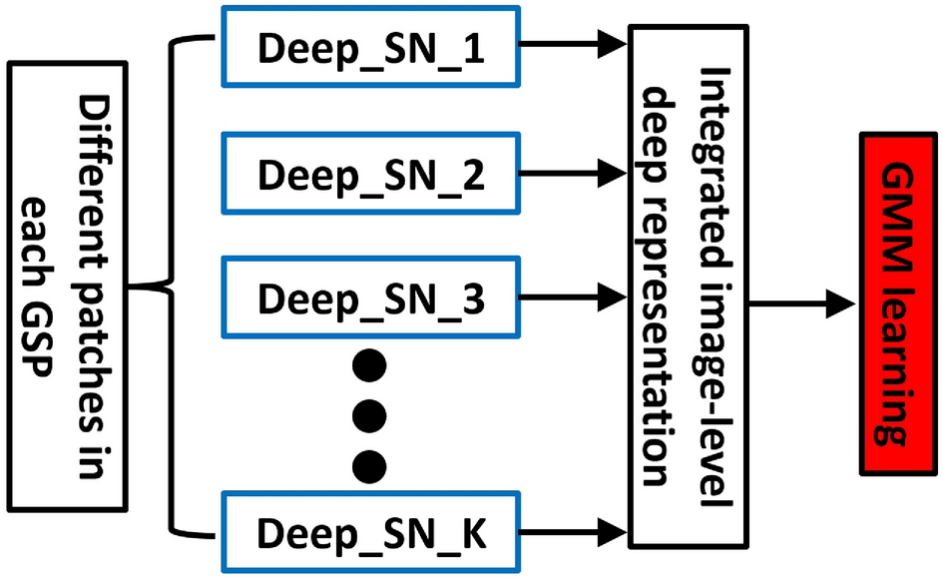
An elaboration of encode GSP into our deep aggregation model (SN: sub network).

Utilizing this approach, we methodically select *L* scenic patches for each scenic image that represent the human GSP, as depicted in Fig. 2. Notably, the initial scenic patch is interactively chosen by the user, aligning with the observation that the human visual system often initially focuses on the central patch of each scenic image. Consequently, we designate the first patch as the central one in every scenic picture, typically positioning it at the core of the scene. For every GSP, its deep representation is calculated in the following.

#### Deep aggregation model for GSP representation

The Binarized Normed Gradients (BING) methodology ^1^ is utilized to identify *K* critical patches for each face based on their visual and semantic prominence, which are then connected to create what is termed a Gaze Shift Path (GSP). Upon establishing a GSP for every face, we construct a sophisticated deep learning model known as the deep aggregation network. This model combines two key elements: 1) A Convolutional Neural Network (CNN) equipped with Adaptive Spatial Pooling (ASP) to conduct an exhaustive analysis of distinct regions, and 2) A mechanism for amalgamating visual inputs from varied regions into a comprehensive feature representation on the image scale.

**Component 1:** The accurate portrayal of spatial attributes of a scene necessitates the maintenance of the original dimensions and shapes of the image ^52^, as non-rectangular object shapes offer deeper insights into scene complexities ^51^. Aiming at this, we adapt a standard hierarchical CNN framework ^54^ to accept inputs of diverse dimensions and shapes by incorporating an ASP layer ^52^ that aligns pooling dimensions with the input shape, thereby ensuring an authentic depiction of the scene.

This CNN analyzes an ensemble of chosen scene patches, introducing variety via random modifications and orientations. The architecture progresses through convolution, ASP, and normalization phases, concluding with a fully connected layer. This design produces a layer that depicts *H* specific patches, utilizing common lower layers to diminish parameter count while preserving vital low-level features.

**Component 2:** For each GSP, we derive an exhaustive, multi-faceted feature set for every patch using the regional CNN mentioned earlier. These features are then unified into an elaborate descriptor for the GSP, effectively integrating the visual information into a cohesive image-level feature.

Let $\Psi =\{\psi _i\}_{i\in [1,K]}$ denote the set of deep features corresponding to each region along a specified path, where each feature vector $\psi _i$ belongs to the space $\mathbb {R}^M$. For each feature component *m*, we compile a set $\mathcal {S}_m$ comprising the *m*-th element from every $\psi _i$, thus forming $\mathcal {S}_m=\{\psi _{mj}\}_{j\in [1,K]}$. To aggregate these features into a unified representation, we utilize a collection of statistical operations $\Pi =\{\pi _u\}_{u\in [1,U]}$, which includes calculations such as minimum, maximum, mean, and median, to process the deep features from each region. The results of these operations are then amalgamated into a singular vector via a densely connected layer, culminating in an *L*-dimensional vector that provides a comprehensive depiction of the Gaze Shift Path (GSP). This vector significantly aids in scene classification by offering a nuanced integration of localized and expansive visual components.

18 $$\begin{aligned} \mathcal {F}(\Psi )=\textbf{P}\times (\oplus _{u=1}^U \oplus _{m=1}^M \pi _u(\mathcal {S}_m)), \end{aligned}$$

In this framework, the parameter matrix $\textbf{P}$, defined within the real number space $\mathbb {R}^{L\times UM}$, serves as the repository for parameters in our deep aggregation layer. We set the number of statistical functions, *U*, to four, corresponding to the number of statistical operations applied to our dataset $\mathcal {S}$. This configuration allows $\textbf{P}$ to integrate various statistical analyses into a unified approach. The operation represented by $\oplus$ signifies vector concatenation, which merges *UM*-dimensional vectors into a larger vector, facilitating a thorough analysis.

**Deep Aggregation Model Training Overview:** In this scenario, the parameter matrix $\textbf{P}$, located in the real number space $\mathbb {R}^{L\times UM}$, acts as the storage for the parameters of our deep aggregation layer. The number *U* is fixed at four to match the number of statistical methods utilized on our dataset $\mathcal {S}$. This arrangement enables $\textbf{P}$ to merge different statistical assessments into a single, integrated strategy. The symbol $\oplus$ denotes vector concatenation, allowing the combination of *UM*-dimensional vectors into an expanded vector, ensuring a detailed examination.

### Scenery retargeting via GMM

Utilizing the deep features derived from each Gaze Shift Path (GSP), we can effectively describe each scenic image by its human perceptual attributes. Subsequently, we formulate a probabilistic framework to capture the distribution of these deep GSP features, acquired during training, for the purpose of retargeting future scenic images.

Given that the interpretation of scenic images is inherently subjective, as individuals may perceive the same image differently, our retargeting approach integrates insights from experienced photographers’ visual perception. To achieve this, we utilize a GMM to represent the refined GSP representations during training:

19 $$\begin{aligned} prob(\mu |\Upsilon )=\sum \nolimits _i f_i*k_i(\nu |\alpha _i,\Sigma _i), \end{aligned}$$

In this model, $f_i$ signifies the relevance of the *i*-th component in the Gaussian Mixture Model (GMM); $\nu$ represents the feature associated with the Gaze Shift Path (GSP); while $\alpha _i$ and $\Sigma _i$ are the GMM’s mean and variance respectively. The similarity between chosen GSP features is assessed using the Euclidean distance.

The objective of retargeting scenic images is to render a perception that mirrors the extensive dataset used for training. Upon encountering an unfamiliar scenic image, the first step is to determine its GSP and refine its features. Then, the significance of each image segment is appraised. To avoid the common distortions associated with techniques like triangle mesh shrinking, we adopt a grid-based approach for resizing. The test scenic image is divided into grids of equal size, with the significance of each horizontal grid *g* determined as follows:

20 $$\begin{aligned} \eta _h(g)=\max _{\mu } \hat{prob}(\nu |\Upsilon ), \end{aligned}$$

**Figure 3:**
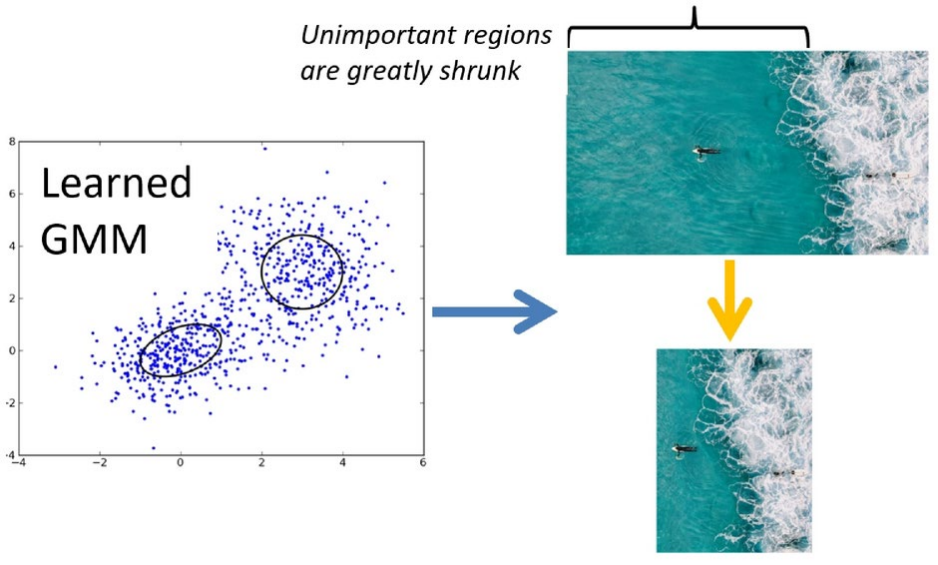
Our proposed grids-guided shrinking method for scenery resizing.

Within this framework, $\hat{prob}$ denotes the probability obtained from the GMM, refined via an Expectation-Maximization (EM) optimization method. The shrinking procedure progresses from left to right (as depicted in Fig. 3), generating an intermediate retargeted iteration of the scenic image at every phase.

Following this, the significance allocated to each horizontal grid undergoes normalization:

21 $$\begin{aligned} \bar{\eta }_h(g_i)=\frac{\eta _h(g_i)}{\sum \nolimits _i \eta _h(g_i)}. \end{aligned}$$

For a scenic picture with dimensions $X\times Y$, the horizontal dimension of each grid $g_i$ is modified to $[X\cdot \bar{\eta }_h(g_i)]$. The vertical significance $\bar{\eta }_v(g_i)$ is determined in a similar manner. As shown in Fig. 3, the clustering results on the left side of Fig. 3 represent the distribution of features derived from the Gaze Shift Path (GSP), which are then used to categorize different regions of the scene. These results show how the features from various grid segments of the scene are clustered into groups based on their relevance to human visual perception. The “Unimportant regions” on the right side of the figure refer to areas of the image that are considered less significant in terms of visual impact. These regions are identified through the clustering analysis, which groups grid sections based on their feature values. The clustering result helps determine which parts of the image are less important for human perception. As indicated in the image, the unimportant regions (those with less relevance or lower GMM probability) are greatly shrunk during the image resizing process, effectively reducing their visual weight in the retargeted scene. The connection between the clustering results and the unimportant regions lies in the GMM’s ability to classify and assess the relevance of different regions. The clustering helps define which regions are less important, and these areas are subsequently shrunk or given less visual prominence in the retargeting process, ensuring that the resized image better reflects the perceptual relevance of each segment.

Using the discussion in this section, the pipeline of the designed scene resizing is provided in Alg. 1.

**Algorithm 1:**
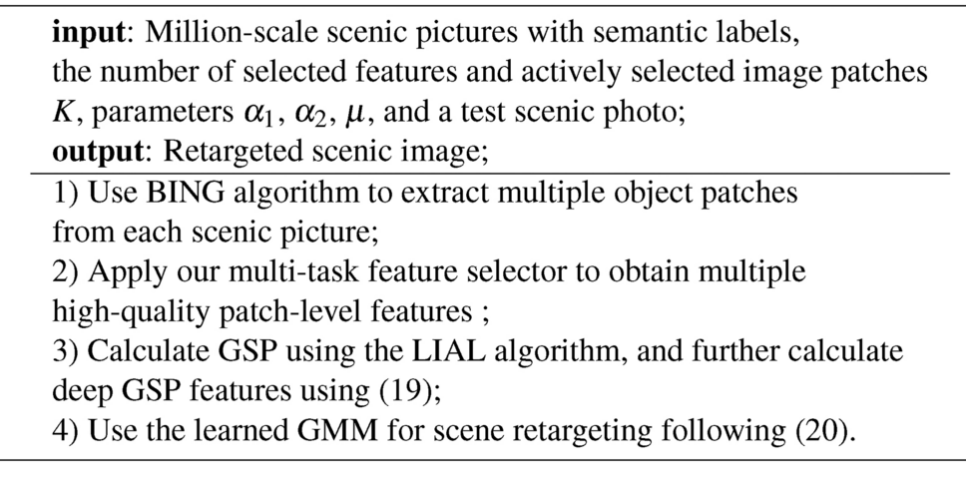
Our Designed Scene Resizing Pipeline

## Empirical assessment

The following datasets are widely used for retargeting and scene recognition. We use them for our experimental evaluation.

- **RetargetMe Dataset**: This dataset is key for studying image retargeting with a focus on human visual perception. It provides images along with gaze data, specifically Gaze Shift Paths (GSPs), which track how individuals engage with different areas of a scene. It allows models to understand perceptual preferences and adjust image resizing to maintain perceptually relevant features ^21^.
- **Scene15 Dataset**: A benchmark collection used for scene classification, containing 15 natural scene categories (e.g., beaches, forests, cityscapes). It is used to train models for classifying images based on their content into one of the 15 categories^39^.
- **Scene67 Dataset**: This dataset includes 67 indoor scene categories (e.g., living rooms, kitchens, offices), enabling the evaluation of scene recognition models specifically for indoor environments^40^.
- **Aerial_ZJU Dataset**: Containing aerial images for recognizing categories such as urban areas, roads, forests, and water bodies, this dataset is designed for developing models to classify aerial images, which is useful for surveillance, land-use mapping, and GIS applications^41^.
- **ImageNet Dataset**: A large-scale image dataset containing over 14 million labeled images across more than 20,000 categories. It is commonly used for training deep learning models, especially convolutional neural networks (CNNs), for image classification tasks^2^.
- **Places Database**: This large-scale scene recognition dataset includes 205 scene categories, providing a diverse set of indoor and outdoor scenes to train deep learning models. It helps models learn meaningful visual features for scene classification tasks^6^.
- **SUN Database**: A comprehensive scene recognition dataset containing over 100 scene categories, ranging from natural environments to constructed spaces. It consists of over 100,000 images and is widely used for evaluating models on large-scale scene classification tasks^42^.

For our experimental setup, we leveraged a powerful server with NVIDIA RTX 3090 GPUs to accelerate deep learning tasks, coupled with an Intel Xeon CPU to handle multi-threaded operations. The system was equipped with 128 GB of RAM to efficiently process large datasets and train complex models without performance issues. To ensure rapid data access and storage, the server included high-speed SSDs. For the model development, we used TensorFlow and PyTorch as our primary frameworks, utilizing CUDA for GPU optimization to maximize computational efficiency. Data processing and manipulation were carried out using Python libraries such as NumPy and Pandas. For training our deep aggregation model, we conducted 120 epochs to achieve the best results.

### Comparative analysis of categorization and retargeting efficiency

Our initial evaluation focuses on the discriminative power of the 128*K* deep Gaze Shift Path (GSP) features. We employ a multi-class Support Vector Machine (SVM) learning strategy as outlined in ^46^. Subsequently, our method is compared against multiple well-known deep visual classification algorithms ^56–62^, noted for their adept encoding of domain-specific knowledge across various scenic categories. We utilize a large-scale dataset of scenic images from ^63^. Public implementations for ^56,57,60,61^ were used directly in our comparative study, adhering to their original settings. For the algorithms in ^58,59,62^, lacking available source codes, we developed custom implementations aiming to match or surpass the performance levels reported in their respective papers.

Furthermore, we contrast our algorithm with various established recognition models, alongside three modern scene classification models ^47–49^. The empirical approaches for our custom-implemented recognition algorithms include: For ^58^, integrating ResDep-128 ^64^ into a multi-label framework, modifying only the fully-connected layer to 19 units while maintaining the original ResDep-128 architecture ^2^. In the case of ^59^, we opt for a ResNet-108 backbone, setting learning rate and decay parameters at 0.001 and 0.05, respectively, and calculating network loss via mean squared error. For ^47^, the well-established object bank ^55^ framework is employed, selecting 18 classes of low-resolution aerial images, using average-pooling, and liblinear for solving the linear classification, with a 10-fold cross-validation for evaluation.

**Table 1:** Compared Outcomes with a Suite of Models (Each test is conducted 15 times, with the standard deviations duly reported).

Category	^56^	^57^	^58^	^59^	^60^	^61^	^62^	SPP+CNN	CleNet
Average	0.651±0.013	0.623±0.011	0.643±0.013	0.651±0.013	0.631±0.014	0.671±0.012	0.671±0.013	0.632±0.015	0.633±0.013
Category	DFB	ML-CRNN	ML-GCN	SSG	MLT	^47^	^48^	^49^	Ours
Average	0.623±0.013	0.656±0.013	0.641±0.014	0.643±0.013	0.621±0.012	0.603±0.011	0.615±0.015	0.623±0.014	**0.683±0.008**

**Table 2:** Computational duration for recognition algorithms under comparison (peak performances are highlighted in bold).

	^56^	^57^	^58^	^59^	^60^	^61^	^62^	SPP-CNN	CleanNet
Train	24h12m	32h11m	43h12m	34h14m	30h25m	40h19m	33h48m	**14h51m**	34h5m
Test	2.231s	2.311s	2.141s	1.872s	3.323s	2.120s	2.311s	1.228s	1.763s
	DFB	ML-CRNN	ML-GCN	SSG	MLT	^47^	^48^	^49^	Ours
Train	31h11m	20h48m	27h16m	40h44m	25h32m	28h43m	32h11m	29h14m	20h13m
Test	1.462s	1.146s	2.535s	1.583s	1.977s	2.241s	2.352s	1.836s	**0.612s**

For the 18 referenced baseline visual recognition algorithms, numerous evaluations were carried out for each, with their average accuracies and associated standard errors (indicated by the symbol “±”) compiled in Table 1. Key findings include: 1) our approach exhibits remarkable competitiveness, especially with respect to per-class standard errors, and 2) our method’s standard deviations are markedly lower than those of the competing algorithms, showcasing superior stability.

### Comparative interactive efficiency

In real-world applications, the duration required for training and testing serves as a vital measure for evaluating the efficiency of a visual classification strategy. As indicated in Table 2, two algorithms demonstrate quicker training times compared to ours, attributed to their simpler and more streamlined designs ^50,65^. Yet, these algorithms fall short by around 4.1% in per-class performance. During the testing phase, our technique exhibits faster execution compared to its alternatives. Given that training occurs offline, the emphasis on reducing testing duration is paramount.

Our scenic image classification framework is structured around three primary elements: 1) the fusion of local and global features, 2) the LIAL method for generating GSPs, and 3) a kernelized classifier for final label assignment. The time expenditure for each phase within the learning process is detailed as: 10 hours 44 minutes for feature fusion, 3 hours 22 minutes for LIAL implementation, and 6 hours 58 minutes for utilizing the kernelized classifier. For the evaluation phase, the time requirements are 232 ms for feature fusion, 317 ms for LIAL, and 68 ms for the kernelized classifier, with the first component consuming a significant portion of the model training time. It’s important to highlight that the extensive training time for the first component can be substantially reduced in practical AI deployments through the use of Nvidia GPUs, potentially achieving up to a tenfold speed increase via program parallelization.

### Comparative analysis of retargeting outcomes

**Figure 4:**
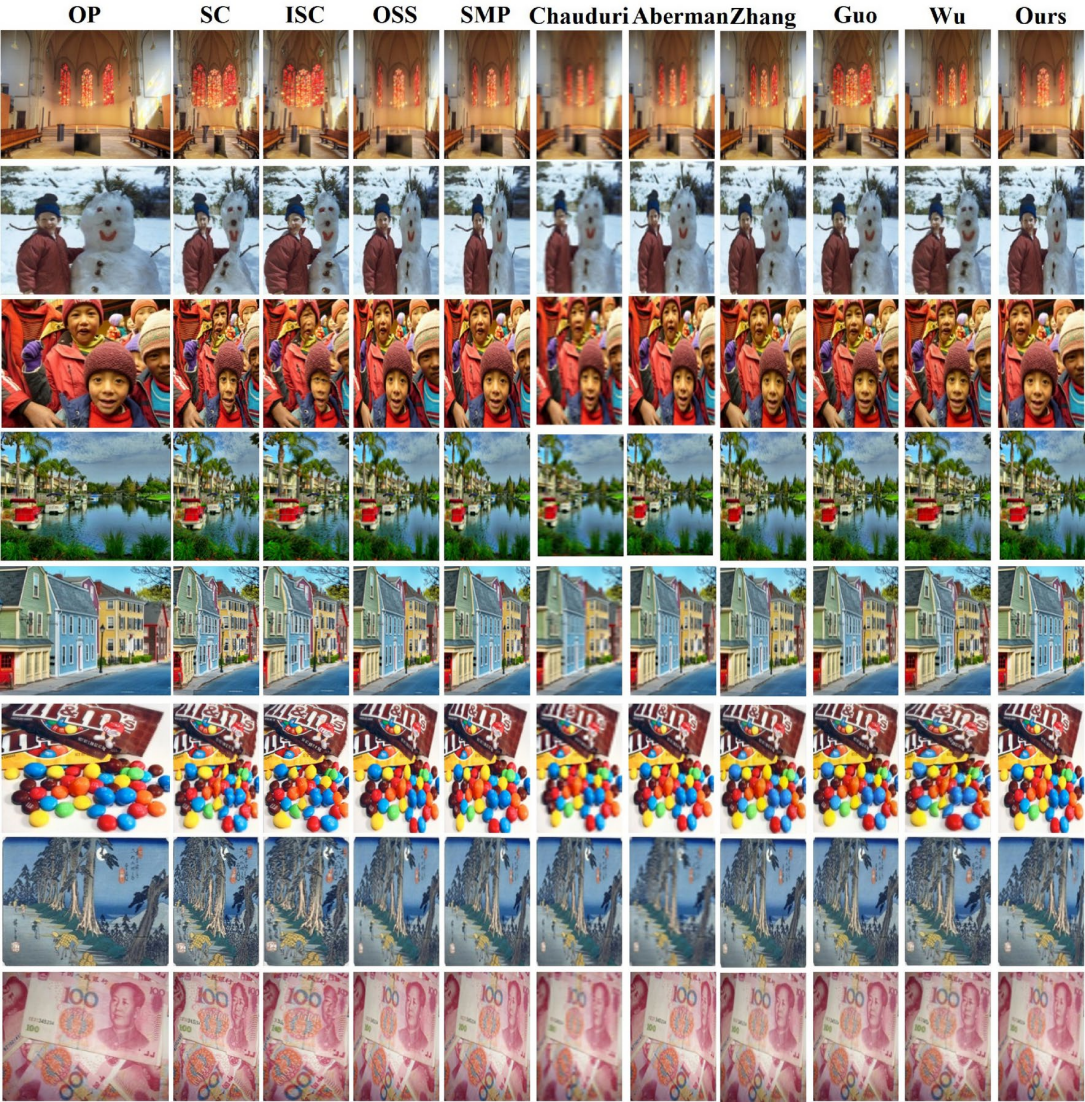
Comparison of various retargeting algorithms using RetargetMet ^21^ photos (OP: original photo).

In our research, we scrutinize the performance of our Gaussian Mixture Model (GMM)-based image retargeting method relative to various recognized image retargeting strategies. This includes a comparison with seam carving (SC) and its enhanced variant (ISC)^9^, the optimized scale and sketch (OSS) method^18^, and the saliency-guided mesh parametrization (SMP) technique ^14^. Figure 4 presents a collection of low-resolution (LR) aerial photo retargeting outcomes achieved by these methodologies. Our approach distinctly excels, yielding retargeted images that are more visually pleasing, successfully preserving the images’ central, semantically significant regions without evident compression and minimizing distortions to boost aesthetic quality. Moreover, two more recent retargeting algorithms are tested. Chaudhuri et al. ^24^ proposed a method that combines joint face detection with facial motion retargeting, targeting multiple faces in an image. Their approach focuses on enhancing the realism of motion transfer between faces, maintaining both the facial expressions and accurate pose adaptation. The method addresses key challenges in multi-face retargeting, demonstrating impressive results in terms of visual quality and accuracy. Aberman et al. ^25^ introduced a skeleton-aware network for deep motion retargeting, a method designed to preserve the skeleton structure of a subject during the motion transfer process. Their network considers the human body’s underlying skeletal structure, enabling more natural and realistic motion adaptation across different body shapes. This approach effectively handles challenges in body motion retargeting, ensuring that both the pose and the animation remain coherent and realistic. Moreover, three very recent deep retargeting models are compared. Zhang et al.^66^ proposed a gaze-guided CT image retargeting method that uses multi-attribute binary hashing to enhance the efficiency of image adjustment. Guo et al.^67^ introduced IRNet-RS, a network that leverages relative saliency for effective image retargeting, improving the preservation of visual content. Wu et al. ^68^ presented a multi-operator approach for retargeting stereoscopic images, focusing on salient feature classification to enhance the image transformation process.

**Figure 5:**
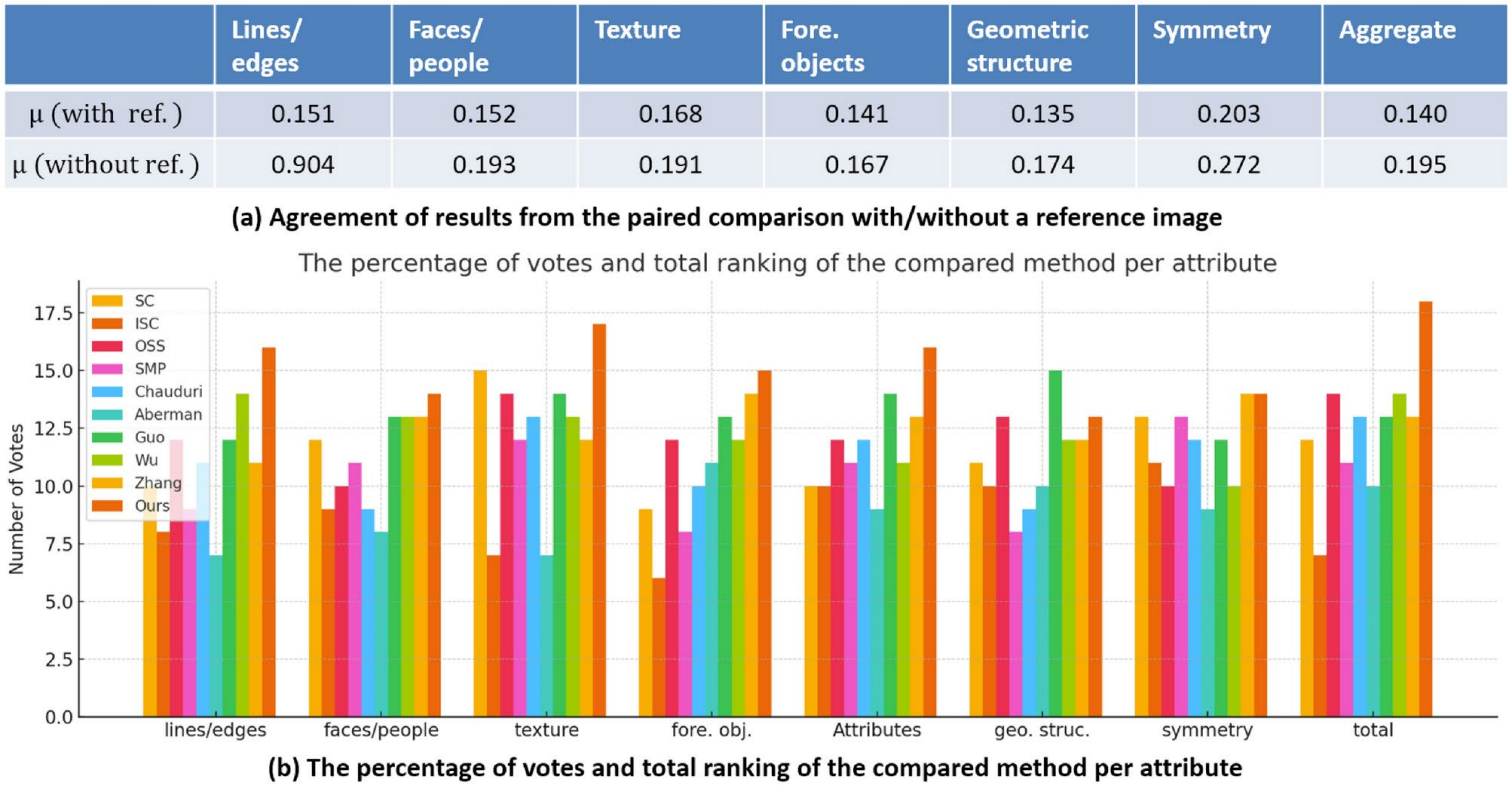
Results of our conducted user study on the compared retargeting algorithms.

A detailed user study was subsequently carried out to further evaluate these retargeting methods. Participating in this study were forty master’s and PhD students from our College of Information Systems. They were tasked with performing aesthetic assessments of two groups of retargeted images: a) comparisons between retargeted and original scenic photographs, and b) evaluations of solely the retargeted images, adopting the empirical framework from ^21^. We employed the agreement coefficient to gauge participants’ preferences towards the retargeted images by the various techniques. A diminished agreement score indicates a higher challenge in discerning the more aesthetically appealing image. As illustrated in Fig.5, the agreement coefficient notably declines when the original scenic images are omitted as a reference. Importantly, participants consistently identified “face/people” and “symmetry” as crucial, underscoring human faces’ significance in visual perception and symmetry as a pivotal aesthetic criterion. In Fig.5b, it’s apparent that our method outperforms competitors, particularly in conserving “face/people” and “texture” features, and slightly surpasses other metrics as well.

### Component-wise performance evaluation

Herein, we evaluate two crucial components of our scenic image categorization framework to verify its efficiency.

**Figure 6:**
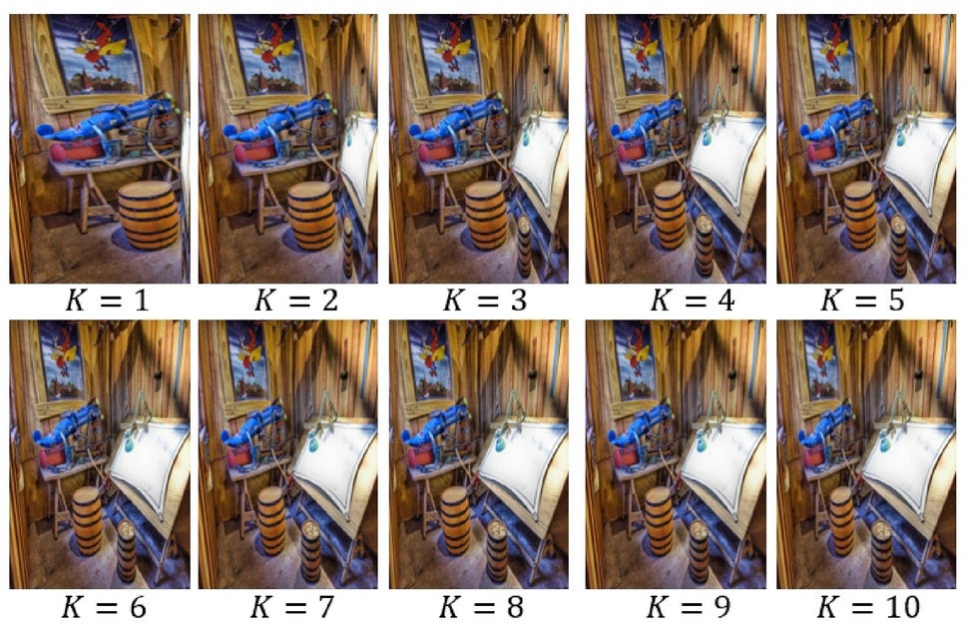
Retargeted scenery images by tuning *K*.

**Figure 7:**
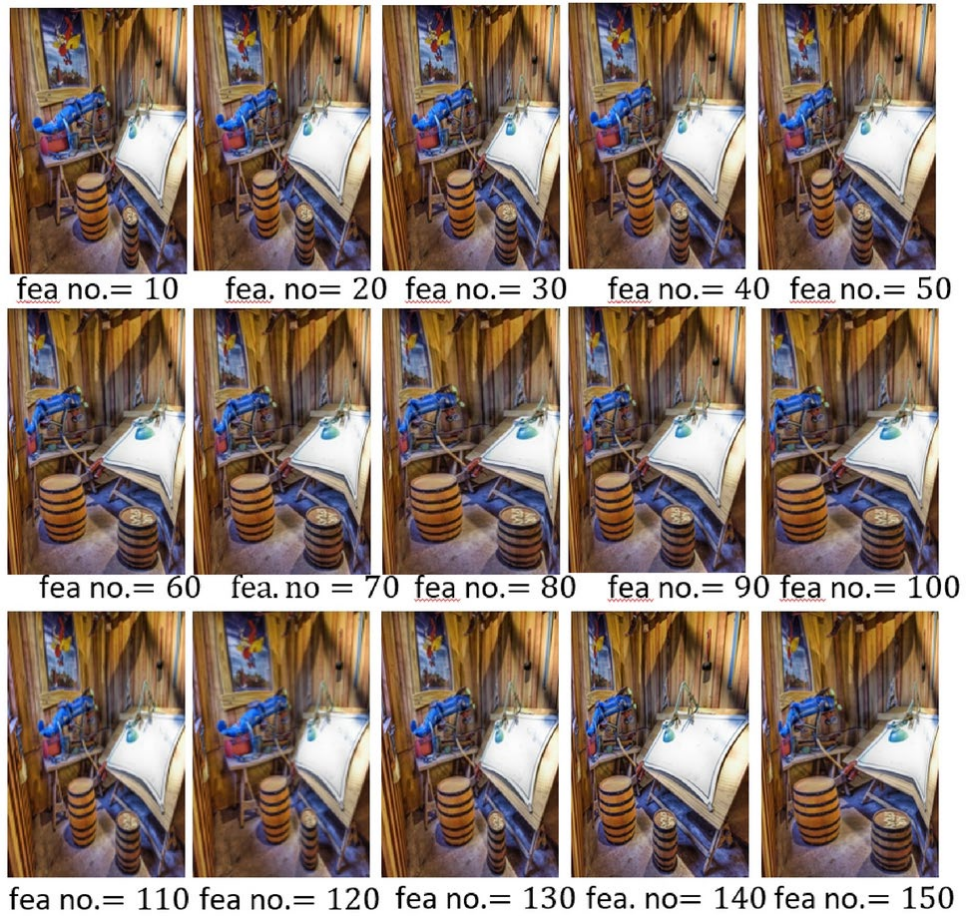
Retargeted scenic images by changing the selected feature number.

Firstly, we analyze the impact of the active learning algorithm embedded in our framework. We remove the active learning feature and instead use a random selection of *K* image patches (referred to as S11) for comparison. We also investigate the effect of using the central *K* patches within each scenic picture (S12), reflecting the natural human inclination to focus on central areas of an image. The performance impact of these modifications is detailed in the second column of Table 3, where both modifications result in a notable decrease in categorization performance, highlighting the usefulness of aligning with human gaze behavior for effective scenic picture encoding. Additionally, in Figs. 6 and 7, retargeting outcomes are presented while adjusting *K* and the dimensionality of patch-level features. Optimal results are observed with $K=5$ and feature dimensionality set to 60.

Subsequently, we assess the effectiveness of our kernelized Gaze Shift Path (GSP) representation for scenic image description across three scenarios. The first scenario (S31) utilizes a multi-layer CNN with aggregation guidance that integrates labels from all patches in a scenic image to assign the final image label. The second (S32) and third (S33) scenarios test the substitution of our linear kernelized feature with polynomial and radial basis function (RBF) kernels, respectively. The alterations in categorization accuracy following these adjustments are detailed in Table 3. Remarkably, employing an aggregation-guided multi-layer CNN (S31) notably reduces the accuracy of scenic image categorization, demonstrating the significance of our selected kernelized GSP representation.

**Table 3:** Performance improvement and reduction through module adjustment (Sij represents the intersection value between column Si and row Oj, for instance, S11 signifies “$-6.322\%$”).

	S1	S2	S3
O1	− 6.323%	− 5.742%	− 6.648%
O2	− 5.244%	− 4.365%	− 3.361%
O3	n/a	− 4.611%	− 1.761%
O4	n/a	− 5.413%	n/a

**Table 4:** Mean accuracy of shallow and deep learning models across six datasets (each test conducted 20 times).

Data set	FLWK	FLTK	MRH	SPM	LLC-SPM	SC-SPM	OB-SPM	SV	SSC
Scene-15	72.1%	74.6%	67.2%	78.6%	82.2%	81.2%	79.6%	82.3%	85.7%
Scene-67	40.9%	41.1%	34.2%	44.3%	48.5%	47.6%	48.2%	49.3%	50.4%
ZJU Aerial	67.5%	68.3%	62.5%	73.2%	78.5%	78.9%	77.6%	79.4%	81.1%
ILSVRC-2010	31.3%	30.7%	27.6%	32.6%	38.4%	36.5%	36.9%	37.4%	38.6%
SUN397	14.9%	15.1%	14.5%	21.2%	39.5%	38.9%	38.4%	39.2%	40.3%
Places205	21.3%	22.4%	20.3%	28.6%	31.3%	32.4%	30.8%	31.6%	32.6%
Data set	ImageNetCNN	RCNN	MCNN	DMCNN	SPPCNN	Ours	FLWK(Saliency)	FLTK (Rectangular)	Mesnil
Scene-15	83.2%	87.4%	87.5%	89.2%	91.5%	**92.3%**	87.4%	88.5%	85.6%
Scene-67	58.6%	62.2%	72.7%	68.3%	65.3%	**75.1%**	71.4%	73.2%	72.3%
ZJU Aerial	75.4%	79.5%	78.5%	80.3%	79.6%	**84.4%**	80.6%	80.3%	79.6%
ILSVRC-2010	36.2%	39.8%	40.5%	41.3%	42.8%	**44.4%**	40.7%	41.2%	40.8%
SUN397	47.4%	48.7%	53.3%	52.3%	53.3%	**56.2%**	51.8%	51.5%	50.9%
Places205	39.3%	44.2%	45.2%	47.5%	49.5%	**52.2%**	48.7%	49.4%	49.8%
Data set	Xiao	Cong	Fast R-CNN	Faster RCNN					
Scene-15	86.7%	86.4%	90.3%	91.5%					
Scene-67	71.9%	72.3%	72.1%	74.3%					
ZJU Aerial	80.3%	80.2%	80.2	81.7%					
ILSVRC-2010	40.6%	40.9%	40.5%	42.3%					
SUN397	50.7%	50.3%	51.7%	52.2%					
Places205	49.4%	48.1%	48.9%	49.8%					

To ascertain the efficacy of our convolutional neural network (CNN) predicated on data aggregation, we instituted experiments under delineated conditions. Initially, we morphed areas of diverse geometries into rectangular patches to ensure complete coverage. This modification led to a decrement in scene categorization accuracy by 6.33%, 3.42%, 4.32%, 2.55%, 3.41%, and 2.24% across the Scene-15 ^39^, 67 ^40^, ZJU Aerial ^41^, ILSVRC-2010 ^2^, SUN397 ^42^, and Places205 ^6^ datasets, respectively. This reduction in accuracy underscores the imperative of maintaining the original contour of regions to aptly convey the scenes’ contextual essence. Furthermore, to probe the influence of regional arrangement on the learning efficacy of our deep model, we shuffled the order of the *K* regions and repeated this process 20 times. Specifically, on the ILSVRC-2010 dataset, we observed an average performance diminution of 2.44%, accentuating the significance of a consistent regional sequence for optimal model training. Detailed comparative outcomes are delineated in Table 4. The results clearly show the advantage of our deep aggregation model.

## Conclusions

The challenge of adeptly retargeting scenic images is pivotal in the realm of intelligent systems. Through this research, we’ve forged a visual composition framework for scenic image retargeting. The key strengths of our approach are threefold: (1) the introduction of a cutting-edge multi-task feature selector for enhancing patch-level features, (2) the deployment of a locality-preserved active learning strategy to create Gaze Shift Paths (GSPs) from scenic images of varying resolutions, and (3) the application of a probabilistic retargeting model utilizing Gaussian Mixture Models (GMM). These innovative components render our retargeting process automatic, efficient, and capable of generating visually appealing retargeted scenic images.

However, a potential limitation of our approach is the possibility of the derived GSP not fully aligning with actual human gaze patterns observed in real-world scenarios. Looking ahead, we aim to conduct an extensive user study to compare the GSPs against authentic human gaze sequences. Our goal is to refine the LIAL technique to ensure the GSPs closely mirror the human visual system’s behavior^4,13–15,20^.

## Supplementary Information

Supplementary Information.

## Data Availability

The datasets used and/or analysed during the current study are available from the corresponding author (Sen Wang) on reasonable request. All the Python codes are presented in the supplementary material.
